# Constitutive overexpression of soybean plasma membrane intrinsic protein GmPIP1;6 confers salt tolerance

**DOI:** 10.1186/1471-2229-14-181

**Published:** 2014-07-07

**Authors:** Lian Zhou, Chuang Wang, Ruifang Liu, Qiang Han, Rebecca K Vandeleur, Juan Du, Steven Tyerman, Huixia Shou

**Affiliations:** 1 State Key Laboratory of Plant Physiology and Biochemistry, College of Life Sciences, Zhejiang University, Hangzhou 310058, P. R. China; 2 Present Address: College of Agriculture and Biotechnology, Southwest University, 400715 Chongqing, P. R. China; 3 Australian Research Council Centre of Excellence in Plant Energy Biology, School of Agriculture, Food and Wine, Waite Research Institute, University of Adelaide, PMB1, Glen Osmond, SA 5064, Australia

**Keywords:** Soybean, Aquaporins, Salt tolerance, Ovexpression, Transformation, *GmPIP1;6*

## Abstract

**Background:**

Under saline conditions, plant growth is depressed via osmotic stress and salt can accumulate in leaves leading to further depression of growth due to reduced photosynthesis and gas exchange. Aquaporins are proposed to have a major role in growth of plants via their impact on root water uptake and leaf gas exchange. In this study, soybean plasma membrane intrinsic protein 1;6 (GmPIP1;6) was constitutively overexpressed to evaluate the function of GmPIP1;6 in growth regulation and salt tolerance in soybean.

**Results:**

*GmPIP1;6* is highly expressed in roots as well as reproductive tissues and the protein targeted to the plasma membrane in onion epidermis. Treatment with 100 mM NaCl resulted in reduced expression initially, then after 3 days the expression was increased in root and leaves. The effects of constitutive overexpression of *GmPIP1;6* in soybean was examined under normal and salt stress conditions. Overexpression in 2 independent lines resulted in enhanced leaf gas exchange, but not growth under normal conditions compared to wild type (WT). With 100 mM NaCl, net assimilation was much higher in the *GmPIP1;6*-Oe and growth was enhanced relative to WT. *GmPIP1;6*-Oe plants did not have higher root hydraulic conductance (*L*_o_) under normal conditions, but were able to maintain *L*_o_ under saline conditions compared to WT which decreased *L*_o_. *GmPIP1;6*-Oe lines grown in the field had increased yield resulting mainly from increased seed size.

**Conclusions:**

The general impact of overexpression of *GmPIP1;6* suggests that it may be a multifunctional aquaporin involved in root water transport, photosynthesis and seed loading. *GmPIP1;6* is a valuable gene for genetic engineering to improve soybean yield and salt tolerance.

## Background

A significant proportion of cultivated land is salt affected representing about 2% of dry-land and 20% of irrigated agriculture (FAO Land and Plant Nutrition Management service, http://www.fao.org/nr/aboutnr/nrl/en/). Soil salinity arises from natural or human-induced processes that inhibits plant growth via osmotically induced water deficit and/or ion toxicity if excessive sodium (Na^+^) and chloride (Cl^−^) accumulate in the shoot via transpiration [1]. Osmotic stress reduces the ability of the plant to extract water from the soil and growth will reduce rapidly and significantly as salt concentration around the roots increases past a threshold level. Ion toxicity occurs when salt (Na^+^ and Cl^−^ ) gains entry via the transpiration stream and accumulates in the shoot to toxic concentrations resulting in injury to cells and causing further reductions in growth [1,2]. Salt tolerance/sensitivity is indicated by the relative degree of biomass reduction in saline soil compared to plants in a non-saline soil, over an extended period of time [3]. Clearly water flow is linked to both types of stresses induced by salinity, yet the role of water transport in plant salt tolerance is not yet clearly defined.

Plants have evolved three distinct mechanisms of salinity tolerance including osmotic adjustment to allow turgor to be maintained, Na^+^ and Cl^−^ exclusion from leaf blades, and compartmentalization of Na^+^ and Cl^−^ at cellular or intracellular sites [1]. Numerous transporters have been identified as likely to be involved in Na^+^ and Cl^−^ exclusion and compartmentation [1,2,4-7], but the proteins that transport water across membranes, the aquaporins, are not considered to be directly involved in these processes, though indirect effects could occur through their impact on osmotically driven water flow and pathways for water and solute flow in roots and leaves [8].

The radial flow of water from soil solution toward the root xylem encounters a relatively high resistance compared to subsequent axial flow in the xylem to the shoot. The radial flow pathway in the root consists of the apoplastic pathway along the intracellular spaces and the cell-to-cell pathway, in which water moves through plasmodesmata or across membranes [9]. Apoplastic water flow can be blocked by Casparian bands and suberin lamellae at key cellular barriers such as the endo and exodermis [10,11] where water transport across membranes occurs. Depending on the plant species and conditions, as well as the position along the root, there are variable contributions of the apoplast pathway compared to the cell-to-cell pathway [8]. The conductance of the cell-to-cell pathway can be largely determined by the activity of aquaporins (AQPs) [12]. AQPs are suggested to play a key role in plant water balance and water use efficiency [8,13-17].

Aquaporins are members of the major intrinsic protein (MIP) family, which in plants are divided into five subfamilies that include the plasma membrane intrinsic proteins (PIPs). These are considered as the main water transport pathway across plasma membranes in root and leaf tissues that play important roles in plant water relations [8,16-19]. According to the N terminal length of the proteins, the PIPs are further divided into two subclasses (PIP1 and PIP2). PIP1s require co-expression of PIP2s to show high water permeability in *Xenopus laevis* oocytes [20-27]. PIP1s and PIP2s interact affecting targeting to the plasma membrane [20,21] and forming hetero-tetramers of variable stoichiometry that appears to affect their transport efficiency [27]. Plant genomes have variable numbers of aquaporin genes, ranging from 35 in *Arabidopsis thaliana*[28], 33 in *Oryza sativa*[29] and 66 in soybean, including 22 *PIPs*[30]. Compared with other species, little is known about the function of *AQP* genes in soybean.

Aquaporins are clearly involved in water transport in roots and leaves [8] and have been linked to water uptake required for cell expansion [18,26,31-35]. Water is the carrier of Na^+^ and Cl^−^ in the transpiration stream contributing to shoot ion toxicity, and in salinity-induced osmotic stress, free energy gradients need to be developed to drive water diffusion to the sites of cell expansion. In this context aquaporins could affect the root’s ion selectivity by determining the proportion of water that flows via membrane pathways relative to the apoplast, while in osmotic stress, they could allow continued water supply under diminished osmotic and pressure gradients by increasing membrane hydraulic conductivity.

Abiotic stresses such as salt, drought and cold influence the water balance of plants and the expression of *AQP* genes [36]. Overexpression of several *AQP* genes in plants confers abiotic stress resistance. Overexpressing *NtAQP1* in tobacco increased photosynthetic rate, water use efficiency and yield under salt stress [17]. Overexpression of several wheat *AQPs*, including *TaNIP*, *TaAQP8* and *TaAQP7* genes in *Arabidopsis* or tobacco also increased salt tolerance or drought tolerance of the transgenic plants [37-39]. Recently, overexpression a *MusaPIP1;2* in banana displayed high tolerance to multiple abiotic stresses including salt, cold and drought [40].

Soybean is a major source of protein and oil for humans and animals, yet relatively mild salt stress significantly reduces soybean growth, nodulation, seed quality and yield [41]. Recently it was found that the expression of *GmPIP1;6* in roots correlated with rapid and longer term changes in root *L*_o_ in response to shoot treatments and appeared to be more concentrated in stellar tissue [42]. These results indicated that GmPIP1;6 may be important in the control of root water transport particularly in response to shoot signals. In this study, *GmPIP1;6* was cloned and functionally characterized. Overexpression of *GmPIP1;6* significantly increased salt tolerance of soybean by improving root *L*_o_ and Na^+^ exclusion.

## Results

### Subcellular localization of GmPIP1;6

**Figure 1:**
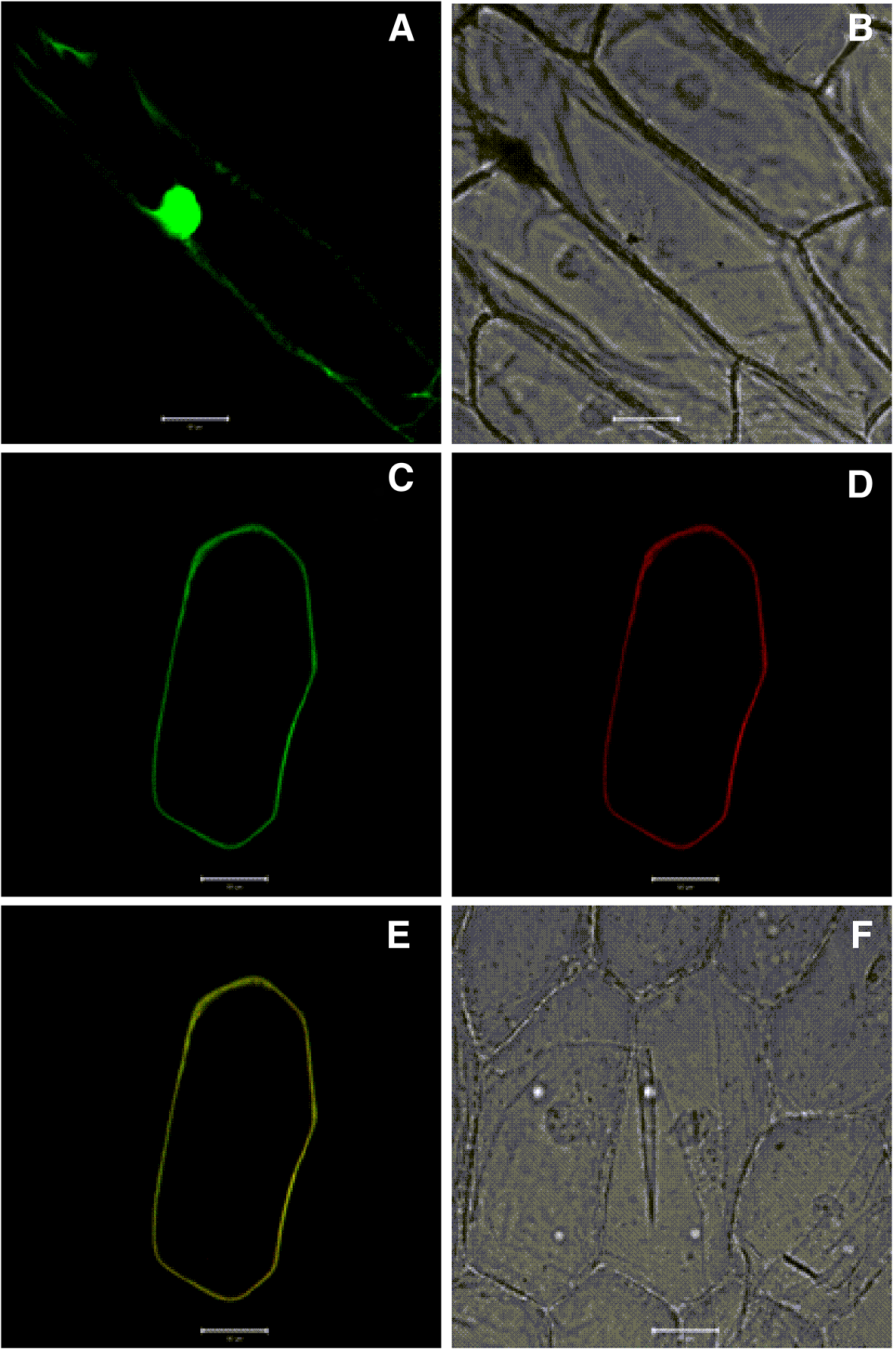
**Subcellular localization of GmPIP1;6. (A)** Green fluorescence image of an epidermal cell expressing the pCAMBIA1302 that sGFP was under the control of the CaMV 35S promoter. **(B)** Bright-field light image of an epidermal cell expressing the pCAMBIA1302. **(C)** Green fluorescence image of an epidermal cell expressing the GmPIP1;6:sGFP fusion protein. **(D)** Red fluorescence image of an epidermal cell expressing the CD3-1007 marker. **(E)** Merged fluorescence image of an epidermal cell expressing the GmPIP1;6:sGFP fusion protein and CD3-1007 marker. **(F)** Bright-field light image of an epidermal cell expressing the GmPIP1;6:sGFP fusion protein and CD3-1007 marker. Bars = 50 μm.

In soybean, *GmPIP1;6* was proposed to be one of the major water transporter genes in roots [42]. The full-length cDNA of *GmPIP1;6* (Phytozome No. Gm08g01860.1) was amplified from soybean roots cultivar Williams 82 by PCR. The cDNA of *GmPIP1;6* is comprised of 1128 bp with an 870 bp open reading frame. The *GmPIP1;6* belongs to the PIP1 subgroup and has an orthologous gene, *GmPIP1;5*, in the soybean genome (Additional file 1: Figure S1). GmPIP1;6 protein contains the characteristic motifs of PIPs and is predicted to be localized on the plasma membrane. To verify the subcellular localization of GmPIP1;6, the GmPIP1;6 was fused with green fluorescent protein (GFP) and driven by a constitutive Cauliflower mosaic virus 35S promoter (CaMV 35S). The final construct GmPIP1;6::GFP was transiently co-expressed in onion epidermal cells and compared with the mCherry plasma membrane marker (Figure 1). GFP fluorescence of GmPIP1;6 was confined to the plasma membrane and co-localized with the RFP fluorescence of mCherry. When the control construct with GFP alone was transiently expressed in onion epidermal cells, the GFP fluorescence was observed in the nucleus and cytoplasm (Figure 1).

### Expression patterns of *GmPIP1;6*

**Figure 2:**
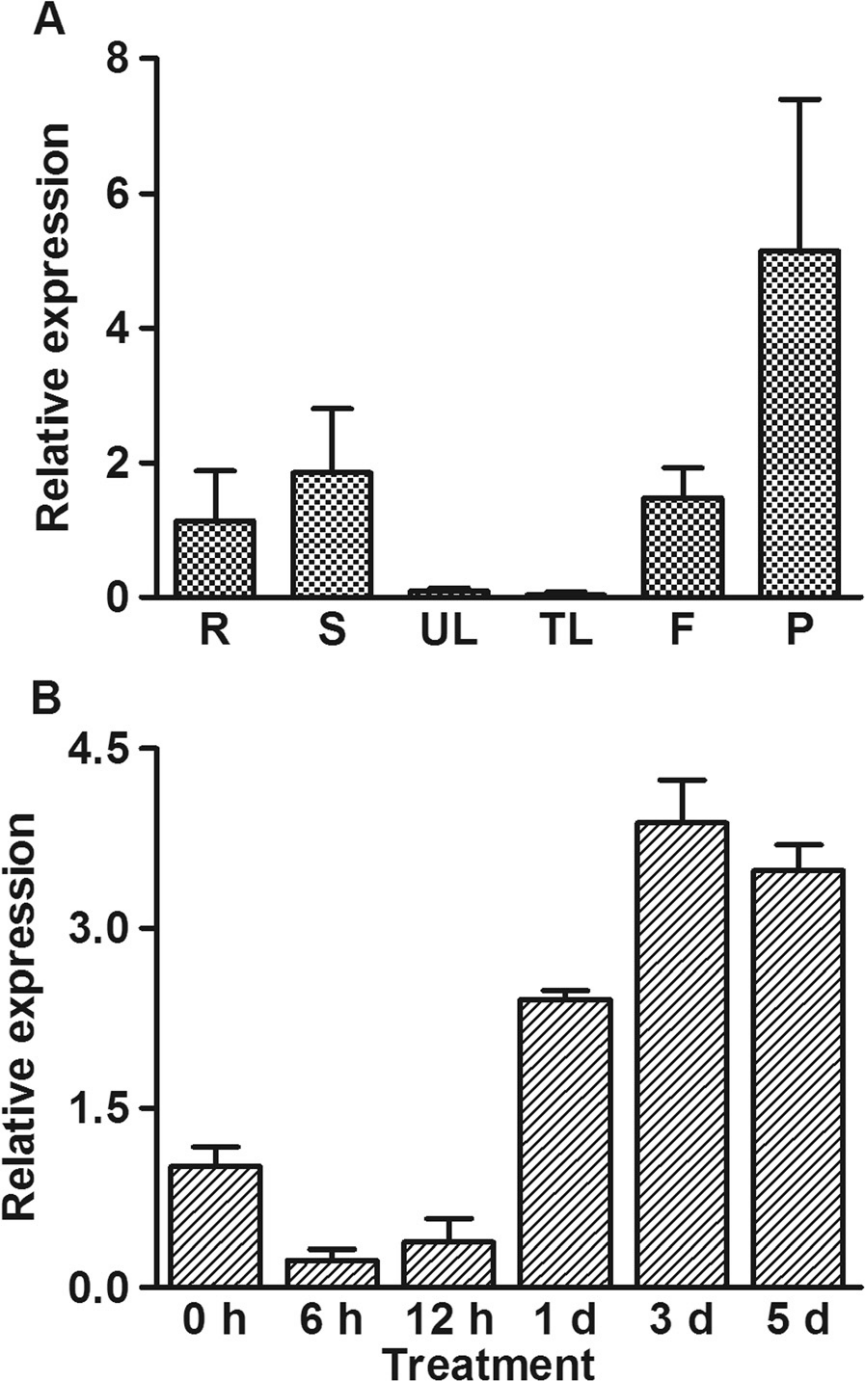
**Expression pattern of *GmPIP1;6 *under normal and NaCl treatments. (A)** Relative expression levels of *GmPIP1;6* in unifoliate leaf (UL), trifoliolate leaf (TL), stem (S), flower (F), pod (P) and root (R). Soybean seedlings were grown in nutrient solution. Total RNA was extracted from different tissues for qRT-PCR. **(B)** Ten-day-old soybean seedlings were treated with or without 100 mM NaCl in nutrient solution. RNA was extracted from the leaves of these seedlings at 6 hours, 12 hours, 1 day, 3 days, 5 days after treatment. All data are means of four biological replicates with error bars indicating SD. Expression level of treated plants was relative to control plants at each time point.

To analyze *GmPIP1;6* gene expression in different soybean tissues, we measured the expression of *GmPIP1;6* in root, stem, unifoliolate leaf, trifoliolate leaf, flower and pod by quantitative RT-PCR. This showed that *GmPIP1;6* was highly expressed in root, stem, flower and pod whereas it was lowly expressed in leaves (Figure 2A). To investigate the response of *GmPIP1;6* to salt stress, we determined expression of *GmPIP1;6* in root and leaf after 100 mM NaCl treatment for 6 hours, 12 hours, 1 day, 3 days and 5 days. The expression of *GmPIP1;6* was suppressed by NaCl treatment in 6 and 12 hours in both roots and leaves. However, expression was induced in the roots after 1 day and further increased at 3 days and 5 days of NaCl treatment (Figure 2B). A similar response was observed in the leaves although the absolute expression of *GmPIP1;6* was much lower than that of the roots (Additional file 1: Figure S2).

### Generation of transgenic soybean overexpressing *GmPIP1;6*

**Figure 3:**
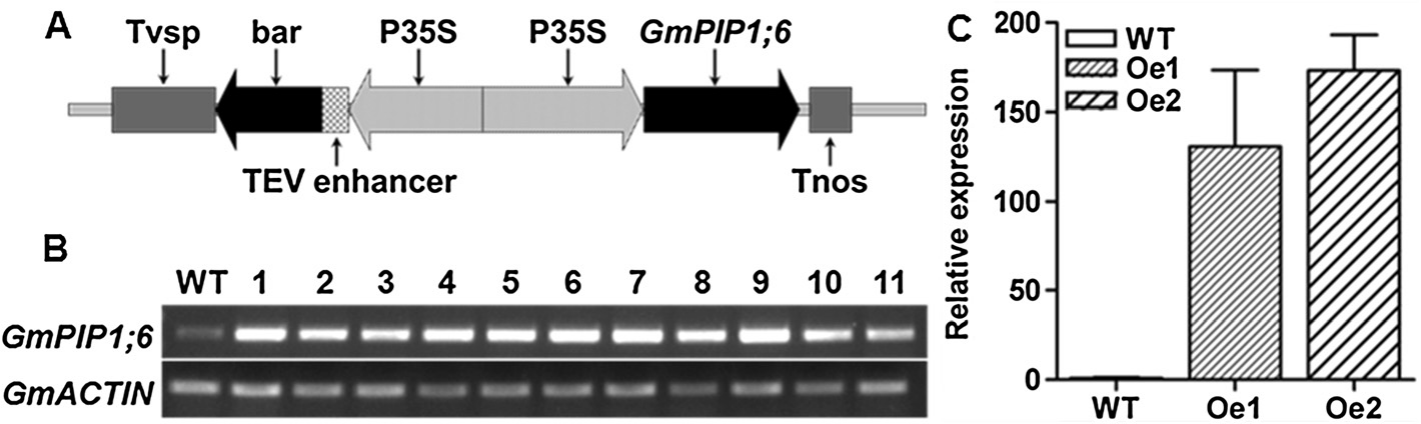
**Construction of *GmPIP1;6 *overexpression transgenic soybean. (A)** Schematic illustration of T-DNA sequence of *GmPIP1;6* overexpression vector. **(B)** Reverse transcript PCR analysis of *GmPIP1;6* overexpression transgenic lines. 1 to 11 represented 11 independent T_1_ generation *GmPIP1;6* overexpression lines. WT, wild type. **(C)** qRT-PCR analysis of two representative *GmPIP1;6* overexpression transgenic lines. RNA was extracted from the leaves of fourteen-day-old seedlings. All data are means of four biological replicates with error bars indicating SD. Expression of *GmACTIN* was used as the internal control.

To characterize the role of *GmPIP1;6* in salt stress, the cDNA of *GmPIP1;6* driven by a modified CaMV 35S promoter was introduced into soybean via soybean cotyledonary node transformation system (Figure 3A). Positive transgenic lines were selected by spraying the herbicide Liberty (Additional file 1: Figure S3). A total of 11 independent lines which overexpressed *GmPIP1;6* were generated and confirmed by semi-qRT PCR (Figure 3B). Two transgenic lines were selected and measured by qRT-PCR. These two lines, which showed more than 100-fold higher expression levels of *GmPIP1;6* than WT control in leaves (Figure 3C), were selected for further experiments.

### Overexpression of *GmPIP1;6* enhances salt tolerance in soybean

**Figure 4:**
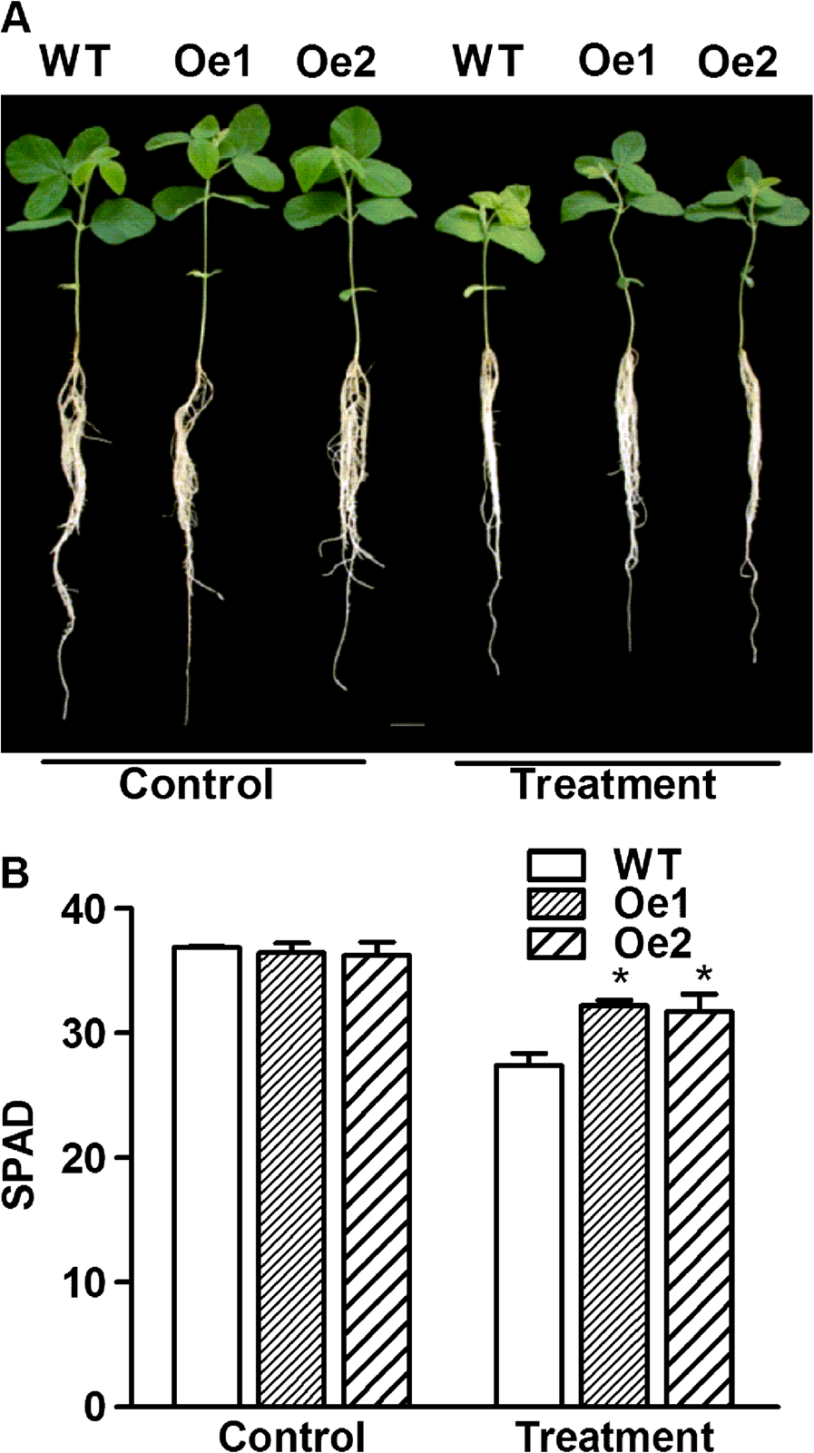
**Salt stress tolerance of *GmPIP1;6 *overexpression transgenic lines.** Ten-day-old WT and *GmPIP1;6*-Oe transgenic soybean plants were treated with or without 100 mM NaCl in nutrient solution for 7 days. **(A)** Photographs of WT and *GmPIP1;6*-Oe transgenic soybean plants with or without 100 mM NaCl treatment for 7 days. Bar = 3.5 cm. **(B)** SPAD of unifoliolate leaf in WT and *GmPIP1;6*-Oe with or without 100 mM NaCl treatment for 7 days. All data are means of four biological replicates with error bars indicating SD.

The growth of WT and *GmPIP1;6*-Oe transgenic soybean plants were similar when grown in aerated hydroponic solution (Figure 4A, Table 1). For salt tolerance analysis, 10-day-old WT and *GmPIP1;6*-Oe transgenic seedlings were treated with 100 mM NaCl for 7 days. Salt treatment suppressed the growth of WT and *GmPIP1;6*-Oe transgenic soybean plants, all of which exhibited a decreased plant length and fresh weight in both leaves and roots (Figure 4A, Table 1). However, the leaves of WT plants turned yellow after treatment for 7 days while the leaves of transgenic plants were still green (Figure 4A). The relative measure of leaf greenness was carried out with a portable chlorophyll meter. Soil-plant analyser development (SPAD) values of unifoliolate leaf in *GmPIP1;6*-Oe were significantly higher than WT under salt stressed condition (Figure 4B). Moreover, the shoot length and fresh weight of *GmPIP1;6*-Oe transgenic soybean were significantly higher than that of WT plants under salt stressed conditions (Table 1). These results indicated that *GmPIP1;6*-Oe plants were more tolerant to salt stress than WT plants.

**Table 1:** Plant length and biomass of the WT and transgenic plants under normal and salt stress conditions

**Genotype**	**Plant length (cm)**		**Plant fresh weight (g)**	
	**Shoot**	**Root**	**Shoot**	**Root**
Normal condition				
WT	22.2 ± 1.5^ab^	38.0 ± 2.1^a^	3.10 ± 0.13^a^	1.15 ± 0.09^a^
Oe1	22.8 ± 0.8^a^	38.2 ± 2.5^a^	3.08 ± 0.08^a^	1.16 ± 0.11^a^
Oe2	22.7 ± 0.5^a^	37.7 ± 2.2^a^	3.10 ± 0.15^a^	1.15 ± 0.06^a^
Salt stress condition				
WT	15.7 ± 1.0^d^	33.0 ± 4.0^ab^	1.72 ± 0.18^c^	0.67 ± 0.10^b^
Oe1	19.0 ± 1.3^bc^	28.8 ± 3.7^b^	2.39 ± 0.17^b^	0.76 ± 0.10^b^
Oe2	18.5 ± 1.6^c^	29.0 ± 3.1^b^	2.28 ± 0.07^b^	0.71 ± 0.07^b^

Ten-day-old WT and *GmPIP1;6*-Oe transgenic soybean plants were treated with or without 100 mM NaCl for 7 days. Length and fresh weight of shoot and root of WT and transgenic plants were measured. Data are given as means ± SD (n = 6). Different letters indicate significant differences (LSD test, P <0.05).

### Overexpression of *GmPIP1;6* increased photosynthesis and root water conductance in soybean under salt stress conditions

**Figure 5:**
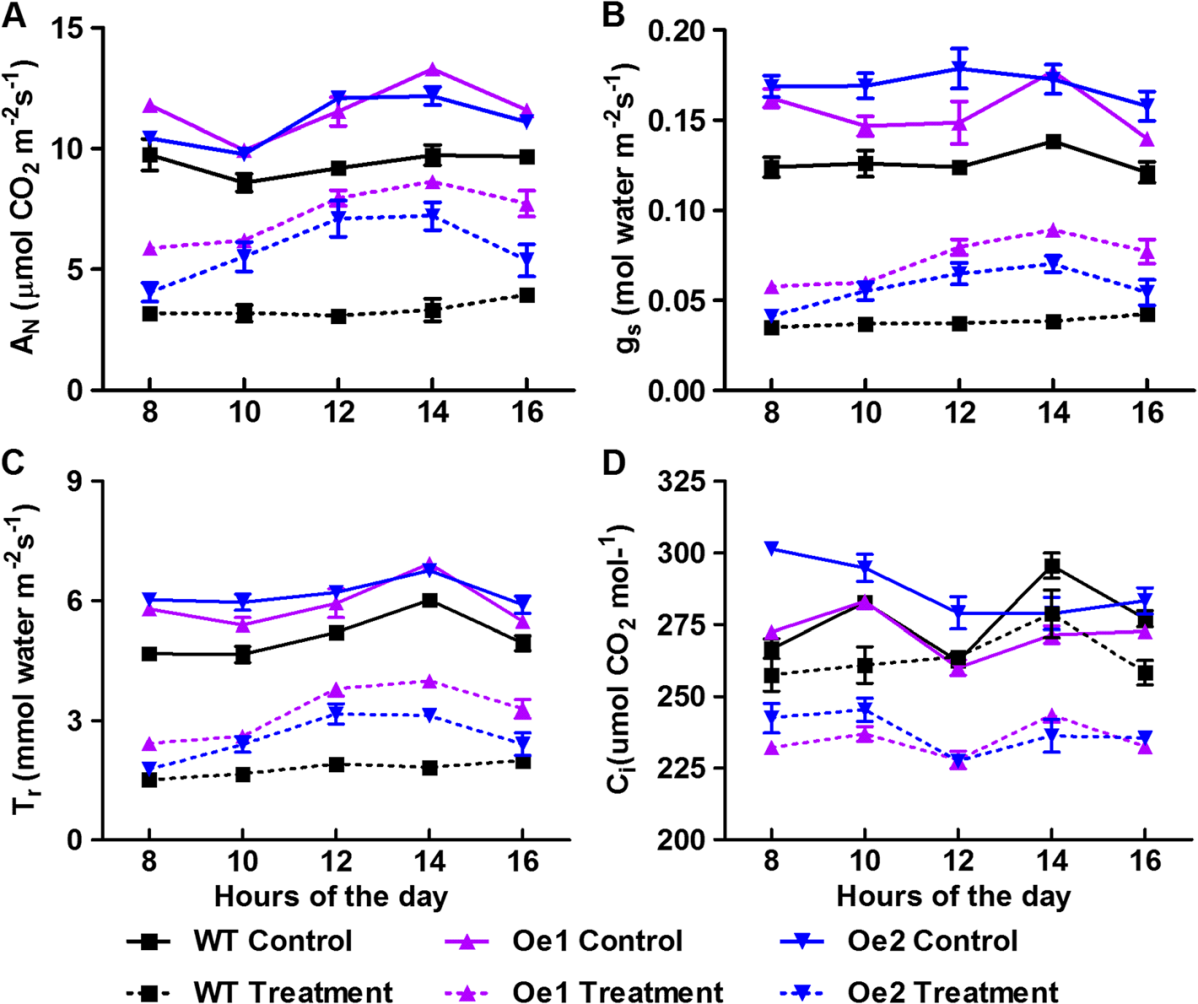
**Daily net assimilation (A**_**N **_**of A), stomata conductance (g**_**s **_**of B) and transpiration rate (T**_**r **_**of C), and substomatal concentration of CO2 (C**_**i **_**of D) of the WT and *GmPIP1;6 *overexpression plants under normal and salt stress conditions.** Ten-day-old WT and GmPIP1;6-Oe transgenic soybean plants were treated with or without 100 mM NaCl for 3 days. A_N_, g_s_, T_r_ and C_i_ were measured by LI-6400 every 2 hours from 8:00 AM to 4:00 PM. All data are means of four biological replicates with error bars indicating SD.

**Figure 6:**
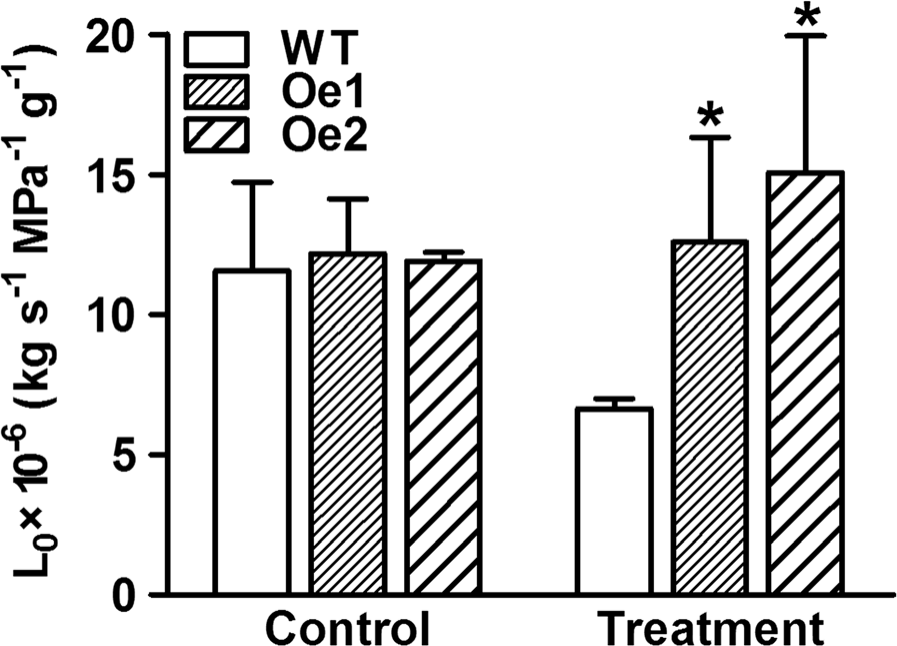
**Root hydraulic conductance (*L***_**o**_**) of WT and *GmPIP1;6 *overexpression plants after salt irrigation.** Five-week-old WT and *GmPIP1;6*-Oe transgenic soybean plants were grown in pot treated with normal irrigation and 50 mM NaCl in a controlled greenhouse. Measurements were made between 10:00 AM to 12:00 AM. *L*_o_ normalized to root dry weight. All data are means of four biological replicates with error bars indicating SD. Asterisks indicate a significant difference between the WT and the two transgenic lines (*P < 0.05).

The impact of *GmPIP1;6* overexpression on net assimilation (A_N_) under saturating light, stomata conductance (g_s_) and transpiration rate (T_r_) were measured using an infrared gas analyser (LI-6400) under normal and salt treatment conditions. Diurnal photosynthesis of soybean was measured every 2 hours in a light period from 8:00 AM to 4:00 PM. As expected, the A_N_, g_s_ and T_r_ showed diurnal changes and peaked at about 2:00 PM in both WT and *GmPIP1;6*-Oe plants (Figure 5A-C). Under normal growth conditions, *GmPIP1;6*-Oe plants showed significantly higher A_N_, g_s_ and T_r_ than that of WT at all the time points measured (Figure 6A-C). We then took the values of A_N_, g_s_ and T_r_ at 2:00 PM to compare WT and *GmPIP1;6*-Oe plants. Under normal growth conditions, A_N_, g_s_ and T_r_ was significantly increased in *GmPIP1;6*-Oe plants than that of WT plants (Table 2). Interestingly, the A_N_, g_s_ and T_r_ was more than 2-fold higher in *GmPIP1;6*-Oe1 plants compared with that of WT under salt treatment. In the other transgenic line, *GmPIP1;6*-Oe2, the A_N_, g_s_ and T_r_ were 1.71, 1.75 and 2.1-fold higher than that of WT (Table 2). These results indicated that overexpression of *GmPIP1;6* increased photosynthetic activity and stomatal conductance, especially under saline conditions.

**Table 2:** Photosynthetic and root hydraulic characteristics of WT and transgenic plants under normal and salt stress conditions

**Variable**	**Normal condition**			**Salt stress condition**		
	**WT**	**Oe1**	**Oe2**	**WT**	**Oe1**	**Oe2**
g_s_ (mol water m^−2^ s^−1^)	0.14 ± 0.01^b^	0.18 ± 0.01^a^	0.17 ± 0.02^a^	0.04 ± 0.01^d^	0.09 ± 0.01^c^	0.07 ± 0.01^c^
T_r_ (mmol water m^−2^ s^−1^)	6.02 ± 0.18^b^	6.93 ± 0.52^a^	6.76 ± 0.34^a^	1.83 ± 0.46^e^	3.99 ± 0.42^c^	3.12 ± 0.53^d^
A_N_ (μmol CO_2_ m^−2^ s^−1^)	9.73 ± 1.26 ^b^	13.29 ± 0.24^a^	12.17 ± 1.12^a^	3.31 ± 1.41^d^	8.26 ± 0.56^bc^	7.21 ± 1.74^c^
C_i_ (μmol CO_2_ mol^−1^)	295 ± 13^a^	271 ± 9^b^	278 ± 17^b^	279 ± 25^d^	243 ± 7^c^	236 ± 17^c^
Stomata pore aperture (μm)	3.48 ± 0.27^b^	3.88 ± 0.42^a^	3.86 ± 0.39^a^	1.93 ± 0.14^d^	3.00 ± 0.25^c^	2.96 ± 0.19^c^
Stomata density (0.1 mm^2^)	19 ± 3^a^	19 ± 3^a^	19 ± 4^a^	19 ± 3^a^	20 ± 4^a^	19 ± 3^a^
IWUE (mmol CO_2_ mmol^−1^ water)	1.61 ± 0.16^c^	1.92 ± 0.15^b^	1.88 ± 0.24^b^	1.73 ± 0.35^bc^	2.17 ± 0.09^a^	2.29 ± 0.22^a^

Ten-day-old WT and *GmPIP1;6*-Oe transgenic soybean plants were treated with or without 100 mM NaCl for 3 days. Data are given as means ± SD (n = 4). Different letters indicate significant differences (LSD test, P <0.05).

Under normal conditions, the substomatal concentration of CO_2_ (C_i_) of *GmPIP1;6*-Oe was lower than that of WT though no significant difference was observed. In contrast, the C_i_ of *GmPIP1;6*-Oe was significantly lower than that of WT under salt treatment (Table 2, Figure 6D). This is in accordance with the higher rate of net photosynthesis of *GmPIP1;6*-Oe compared to WT plants under saline conditions. Instantaneous water use efficiency (IWUE = A/T) was significantly increased in *GmPIP1;6*-Oe plants under both normal and salt stress conditions compared with WT (Table 2). Changing stomata density and/or pore area will influence the g_s_ and T_r_. Examination of the abaxial leaf surface revealed a significantly wider stomatal aperture in *GmPIP1;6*-Oe plants under both normal and salt stress conditions while the stomata density was not changed (Table 2, Additional file 1: Figure S4A). As a result, the water loss rate was increased in the transgenic plants compared with WT plants (Additional file 1: Figure S4B).

We also measured root hydraulic conductance (*L*_o_), normalized to root dry weight, in *GmPIP1;6*-Oe and WT plants. Interestingly the *L*_o_ was similar between *GmPIP1;6*-Oe and WT plants irrigated with nutrient solution. However when irrigated with nutrient solution containing 50 mM NaCl, *L*_o_ of WT plants decreased almost 50% while *L*_o_ of *GmPIP1;6*-Oe plants remained unchanged (Figure 5).

### Overexpression of *GmPIP1;6* affects Na uptake and exclusion of transgenic plants under salt stress

**Figure 7:**
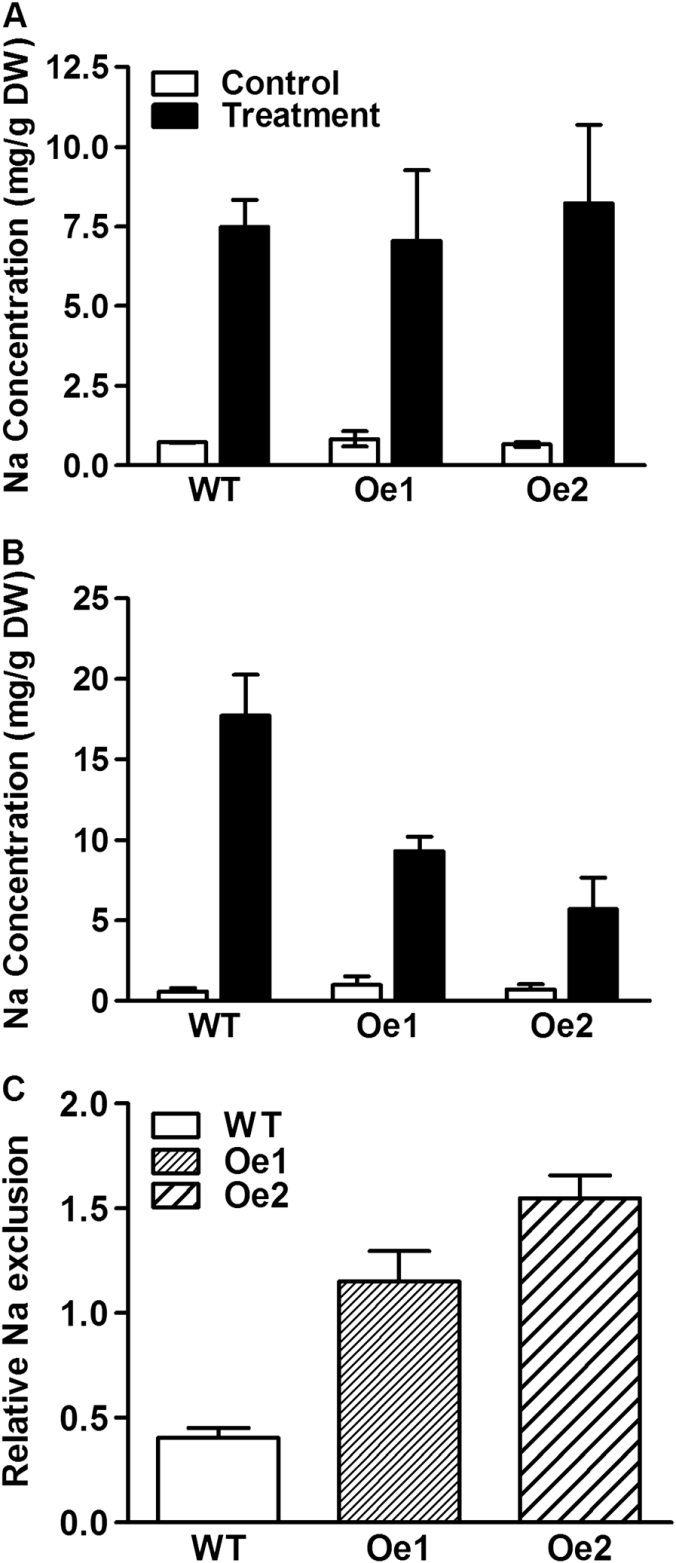
**Ion concentration in WT and *GmPIP1;6 *overexpression plants in hydroponics condition. (A)** and **(B)** The content of Na^+^ in roots and leaves. Ten-day-old WT and *GmPIP1;6*-Oe transgenic soybean plants were treated with or without 100 mM NaCl for 7 days. The roots or leaves of these seedlings were sampled for measurement. **(C)** Relative Na exclusion. Data are means of four biological replicates with error bars indicating SD. Asterisks indicate a significant difference between the WT and the transgenic lines (**P < 0.01). DW, dry weight.

**Figure 8:**
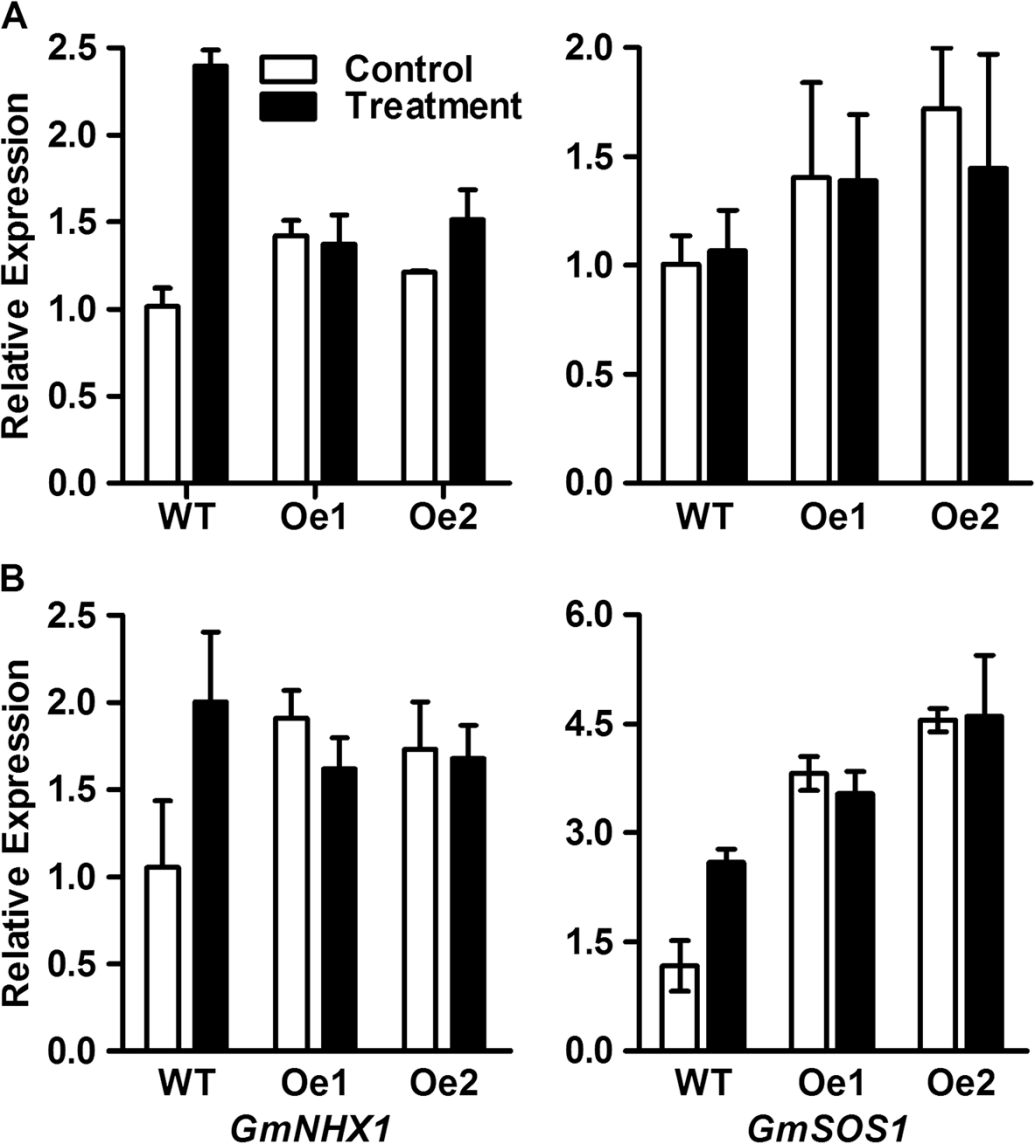
**Expression analysis of soybean salt response gene. (A)** and **(B)** The expression of *GmNHX1* and GmSOS1 in leaves and root under normal or salt stress conditions. RNA was extracted from leaves and roots of 10-day-old WT and *GmPIP1;6* overexpression plants treated with or without 100 mM NaCl for 3 days. All data are means of four biological replicates with error bars indicating SD. Expression of *GmACTIN* was used as the internal control.

The sodium concentration of plants was analyzed under normal and salt stress conditions. Sodium (Na^+^) concentration was similar between WT and *GmPIP1;6*-Oe plants in roots and leaves under normal conditions (Figure 7A, B). Salt treatment increased Na^+^ concentration in the roots and leaves of both WT and *GmPIP1;6*-Oe plants. However, the Na^+^ concentration was significantly lower in the leaves of *GmPIP1;6*-Oe plants than WT plants under salt stress (Figure 7B, Additional file 1: Figure S5). We examined relative Na^+^ exclusion of WT and *GmPIP1;6*-Oe plants after salt treatment revealing that the relative exclusion of Na^+^ from the shoot of *GmPIP1;6*-Oe plants was higher relative to WT (Figure 7C). Moreover, salt treatment induced the expression of *GmNHX1* in the leaves and roots of WT but not in *GmPIP1;6-Oe* plants (Figure 8).

### Overexpression of *GmPIP1;6* increased yields of soybean in the field

Four independent *GmPIP1;6*-Oe lines, where two of these lines were the same as the ones used in the physiology experiments, were grown in field conditions for an entire growing season in two continuous years. Each year, the transgenic plants were compared with WT and null transgenic plants, which were segregated from heterozygous transgenic plants. Interestingly, *GmPIP1;6*-Oe plants have a significantly higher seed weight per plant and per 100 seeds than WT (Table 3). Other yield parameters were similar between WT and *GmPIP1;6*-Oe plants (Table 3, Additional file 1: Figure S6A). A detailed analysis showed that the increased seed weight of *GmPIP1;6*-Oe plants was caused by large seed size (Additional file 1: Figure S6B, Table 3).

**Table 3:** Agronomic characteristics of WT, null transgenic and overexpression transgenic soybean plants in field

**Genotype**	**WT**	**Negative**	**Oe1**	**Oe2**	**Oe3**	**Oe4**
Plant height (cm)	83 ± 3^a^	87 ± 8^a^	83 ± 8^a^	89 ± 10^a^	83 ± 15^a^	87 ± 6^a^
Branch number	4.6 ± 1.1^a^	5.6 ± 2.1^a^	4.7 ± 1.4^a^	4.8 ± 2.1^a^	5.4 ± 2.3^a^	5.0 ± 0.9^a^
Node number/plant	24.6 ± 1.1^a^	24.7 ± 1.1^a^	23.7 ± 2.7^a^	24.3 ± 2.0^a^	23.2 ± 1.6^a^	22.7 ± 2.^4a^
Pod number/plant	175 ± 24^ab^	197 ± 40^ab^	195 ± 18^ab^	174 ± 28^ab^	200 ± 56^a^	166 ± 34^ab^
Seed number/plant	425 ± 62^a^	423 ± 78^a^	435 ± 45^a^	417 ± 69^a^	441 ± 175^a^	406 ± 93^a^
Seed weight (g)/plant	49.6 ± 12.1^b^	47.7 ± 14.2^b^	59.5 ± 6.5^a^	56.7 ± 8.2^a^	58.5 ± 23.0^a^	53.7 ± 12.4^a^
100 seed weight (g)	14.0 ± 0.5^b^	13.6 ± 1.6^b^	16.4 ± 0.8^a^	17.4 ± 1.8^a^	16.8 ± 1.7^a^	16.7 ± 1.0^a^
10 seed length (cm)	7.20 ± 0.07^c^	7.14 ± 0.11^c^	8.02 ± 0.08^a^	7.72 ± 0.08^b^	7.71 ± 0.08^b^	7.80 ± 0.09^b^
10 seed width (cm)	6.28 ± 0.13^c^	6.36 ± 0.11^c^	7.26 ± 0.05^a^	6.90 ± 0.07^b^	7.02 ± 0.08^b^	6.95 ± 0.10^b^

Data are given as means ± SD (n = 6). Different letters indicate significant differences (LSD test, P <0.05).

## Discussion

Recently, 66 *AQP* genes were identified in soybean by a genome wide analysis [30]. The *GmPIP* subfamily contained 8 *PIP1* genes and 14 *PIP2* genes, all of which were predicted to localize on the plasma membrane. It is found that PIP2 aquaporins when expressed in *Xenopus* oocytes have high water permeability while PIP1 aquaporins do not. However, PIP1 aquaporins can work cooperatively with PIP2s in targeting to the plasma membrane and in water permeation as heterotetramers [20-27]. This is accordance with the fact that GmPIP1;6 protein fused with GFP localized on the plasma membrane (Figure 1).

GmPIP1;6 is the ortholog of AtPIP1;2, NtAQP1, HvPIP1;6/1;1 and TaAQP8 in *Arabidopsis*, tobacco, barley and wheat (Additional file 1: Figure S1). AtPIP1;2 and NtAQP1 play a key role in regulating root hydraulic conductance (*L*_o_) in *Arabidopsis* and tobacco [17,43,44], respectively. *In situ* PCR showed that GmPIP1;6 was highly expressed in the stellar region of the root [42], which is similar as NtAQP1. Shoot topping rapidly decreased root hydraulic conductance (*L*_o_) by 50% to 60%, which is correlated with the reduced expression of *GmPIP1;6* in roots of soybean. Therefore, GmPIP1;6 was suggested to control the *L*_o_ as AtPIP1;2 and NtAQP1.

Water stress caused by drought, salt or cold has a complex effect on the expression of *AQP* genes [36]. In summary, the expression of *AQP* genes could be divided into two stages. In the early stress response, the plant usually suppresses the expression of *PIP* genes, which is hypothesised to avoid water flow from the root to the soil when the soil water potential decreases [45,46]. After a few days of acclimation, the expression of *PIP* genes recovers or even increases and is correlated with increased hydraulic conductance [47-49]. The expression of *GmPIP1;6* in both roots and leaves showed this two stage response under salt stress (Figure 2B, Additional file 1: Figure S2), indicating *GmPIP1;6* may be involved in the salt stress acclimation of soybean.

Overexpression of several *PIP1* genes increased the hydraulic conductance and salt tolerance of the transgenic plants, such as *NtAQP1*, *OsPIP1;1*, *TaAQP8* and *MusaPIP1;2*[17,37,40,50]. Here we show that *GmPIP1;6* conferred salt tolerance, but also under normal conditions the overexpression resulted in higher growth and greater yield under field conditions compared to WT plants (Figure 4A, Table 3). However, the mechanism of how these *PIP1* genes can improve plant growth and salt tolerance is largely unknown, though a high K^+^/Na^+^ ratio was mentioned with overexpression of *TaAQP8*[37].

It is highly unlikely that GmPIP1;6 can transport Na^+^, therefore salt tolerance of transgenic *GmPIP1;6* plants is more likely to occur through indirect mechanisms: First, improvement in water uptake by roots and leaf cell hydration, could improve energy capture and conversion by leaves. Greater energy availability in turn could improve Na^+^ exclusion by roots and improve tissue Na^+^ compartmentalization [1]. We compared the root *L*_o_ of WT and *GmPIP1;6-Oe* plants under normal and salt stressed conditions (Figure 7). As expected, NaCl treatment decreased *L*_o_ by 50% in WT plants. In contrast, *GmPIP1;6-Oe* plants maintained *L*_o_ under salt stress conditions. Therefore, *GmPIP1;6-Oe* plants may have a higher water uptake activity than WT plants under saline conditions. Secondly, Na^+^ is transported to shoots in the transpiration stream through the xylem, but it can return to root via the phloem [43,51-53]. Export of Na^+^ from leaves in the phloem could conceivably help to maintain low salt concentration in the leaves and may be enhanced by greater water permeability in phloem cells. Also we show that net assimilation and gas exchange are enhanced in the *GmPIP1;6-Oe* plants, and especially so under saline conditions compared to WT. This would potentially translate to a higher capacity to exclude Na^+^ via energy demanding salt exclusion mechanisms in the roots and the leaves. Thirdly, we measured the expression of *GmNHX1*[54] to analyze the effect of Na^+^ compartmentalization in the vacuole (Figure 8). Salt treatment induced the expression of *GmNHX1* in the leaves and roots of WT but not in *GmPIP1;6-Oe* plants. This is accordance with the lower Na^+^ concentration of *GmPIP1;6-Oe* plants and indicated that vacuole compartmentalization of *GmPIP1;6-Oe* plants was not necessarily enhanced.

Another possibility that may account for reduced Na^+^ transport to the shoot in the over expressing plants could be that more water flow occurs radially across roots via the cell-to-cell (membrane) pathway, as opposed to the apoplast pathway. This would occur because of the higher activity of *GmPIP1;6* in root membranes under salinity stress demonstrated by the higher root *L*_o_ compared to WT. A higher proportion of water flow via the membrane pathway in roots would confer a greater degree of ion selectivity relative to flow in the apoplast pathway. Altogether, we clarified that overexpression of *GmPIP1;6* increased soybean salt tolerance by maintaining water uptake ability and Na^+^ exclusion.

In addition to function as a water channel, AtPIP1;2 and NtAQP1 may function to facilitate CO_2_ transport and enhance photosynthesis by increasing the mesophyll conductance to CO_2_ diffusion [55-59]. Overexpression of *NtAQP1* in tobacco and tomato increased the A_N_, which resulted in increased WUE. The overexpression of *NtAQP1* produced higher dry biomass and yield under normal irrigation and salt stressed conditions [17]. *GmPIP1;6-Oe* plants also exhibited higher A_N_, g_s_ and IWUE than WT under both normal and saline conditions (Figure 6, Table 2). However, the growth of *GmPIP1;6-Oe* plants was only enhanced under saline conditions compared to WT plants (Figure 4A, Table 1). Whether GmPIP1;6 has a similar function as NtAQP1 to facilitate CO_2_ diffusion across leaf cell membranes requires further research.

Importantly, *GmPIP1;6-Oe* plants showed higher yield in the field than WT because the seed weight and size of *GmPIP1;6-Oe* were increased (Table 3, Additional file 1: Figure S6). This may be reflecting the higher net assimilation, but also may indicate sink limitation of seed loading that could be enhanced by greater water permeability in the seed loading process [60]. In addition to being highly expressed in roots and stems, the transcripts of *GmPIP1;6-Oe* were abundant in flower and pod, which supports a role of *GmPIP1;6-Oe* in seed loading of assimilates via enhanced water permeability.

## Conclusions

In this study, the function of *GmPIP1;6* was analyzed by constitutive expressing in the soybean plants. The expression of *GmPIP1;6* was influenced by salt stress. Overexpression of *GmPIP1;6* improved salt tolerance of transgenic plants by increasing water transport, photosynthesis and Na^+^ exclusion. Moreover, the yield of *GmPIP1;6* overexpression plants was improved in the field indicating the potential of *GmPIP1;6* in genetic engineering of soybean.

## Methods

### Plant materials, growth conditions and treatments

Soybean cultivar Williams 82 was used for all physiological experiments and soybean transformation. Seeds were germinated in nursery pots with sand. Five days after germination, the seedlings grown uniformly were transferred into pots with nutrient solution or soil. 1/2 Hoagland solution was used for hydroponic culture containing 2.5 mM KNO_3_, 2.5 mM Ca(NO_3_)_2_, 0.5 mM KH_2_PO_4_, 0.25 mM K_2_SO_4_, 1 mM MgSO_4_, 0.1 mM Fe-EDTA(Na), 4.57 μM MnCl_2_, 3.8 μM ZnSO_4_, 0.09 μM (NH_4_)_6_Mo_7_O_24_, 23 μM H_3_BO_3_, 1.57 μM CuSO_4_. Plants were grown in green house under 12 h light/12 h dark photoperiod with light intensity of 1000 μmol m^−2^ sec^−1^ and day/night temperatures of 30/22°C. Humidity of the growth room was controlled at approximately 30%.

Ten-day-old seedlings were transferred into nutrient solution with or without 100 mM NaCl. The nutrient solution was changed every two days. In the soil experiments, plants were irrigated nutrient solution every three days.

### Subcellular localization of GmPIP1;6

Full length cDNA of *GmPIP1;6* without stop code was amplified via PCR using the primers in Supplementary Additional file 2: Table S1. The PCR product was cloned into vector pCAMBIA1302 under the control of the CaMV 35S promoter. The resulting construct (pCAMBIA1302:*GmPIP1;6*) placed *GmPIP1;6* in-frame, upstream of the sGFP. Plasmids DNA of pCAMBIA1302:*GmPIP1;6* and CD3-1007 (AtPIP2A::mCherry fusion) was mix with 50 μl gold particles and bombarded into onion inner epidermal cells using the Biolistic PDS-1000/He particle delivery system (BIO-RAD). Fluorescence was observed by confocal laser scanning microscopy (LSM700; Carl Zeiss) after incubation at 25°C for 16-18 h on MS medium in dark.

### Construction of transgenic plants

Full-length cDNA of *GmPIP1;6* was amplified by PCR with cDNA of Williams 82 and ligated into pMD-18 T vector (Takara). After sequencing, the correct *GmPIP1;6* was digested from pMD-18 T vector using *Bam*HI and *Xba*I restriction enzymes. *GmPIP1;6* was then cloned into binary plasmid pTF101-35S which was modified by introducing CaMV 35S promoter and nos terminator into pTF101. The vector was transformed into Williams 82 via *Agrobacterium tumefaciens* media soybean cotyledon node transformation system as described [61].

### RNA extraction

Total RNA was isolated from tissues of soybean cultivar Williams 82 using TRIzol reagent (Invitrogen, Carlsbad, CA) according the manufacturer’s instruction. 50 mg soybean tissues with three bilogical replicate were quickly harvested, frozen in liquid nitrogen and stored at -80°C. Contaminating DNA was removed with DNaseI treatment for 20 min at 25°C (Takara), and RNA was stored at -80°C. Total RNA was quantified with nanodrop.

### Semi-quantitative RT-PCR and quantitative real-time PCR

First-strand cDNAs were synthesized from total RNA using SuperScript II reverse transcriptase (Invitrogen). Semi-quantitative RT-PCR was performed using a pair of gene-specific primers. The housekeeping gene *GmACTIN* was used as an internal control. Quantitative real-time PCR was performed using a SYBR Green I on a Light Cycler 480 II machine (Roche Diagnostics), according to the manufacturer’s instructions. The amplification program for SYBR Green I was performed at 94°C for 10 sec, 58°C for 10 sec and 72°C for 10 sec. Triplicate quantitative assays were performed on each cDNA sample. The relative level of expression was calculated using the formula 2 ^-△(△cp)^. All primers used for RT-PCR are given in Supplementary Additional file 2: Table S1.

### Gas-exchange measurements

Homozygous lines were selected from the T_2_ generation of transgenic *GmPIP1;6* overexpression plants and used for the physiology experiment. A_N_, g_s_, T_r_ and C_i_ were recorded in *GmPIP1;6* overexpression and control plants in green house on fully expanded leaves, using an Li-6400 portable gas-exchange system (LI-COR). All measurements were conducted between 8:00 AM and 4:00 PM. Photosynthesis was induced in saturating light (1000 μmol m^−2^ s^−1^) with 400 μmol mol^−1^ CO_2_ surrounding the leaf. The leaf-to-air VPD was kept at around 2 to 4 kPa and leaf temperature was approximately 30°C (ambient temperature) during all measurements. For each treatment, there were four biological replicates.

### Stomata aperture and density

Epidermis of soybean abaxial leaf was separation by forceps. All samples were collected around 2:00 PM (at peak transpiration). Counting and photographing were performed with a bright-field microscope (80i; Nikon) mounted with a camera. Stomata images were later analyzed to count the number per 0.1 mm^2^ area and determine aperture using the microscope software (NIS elements) measurement tool. A microscopic ruler was used for the size calibration.

### Determining Na^+^ concentration

Leaves and root from 17-day old transgenic lines and WT were sampled and dried at 80°C for 3 days. 50 mg of the material was weighed and dissolved with 3 ml of nitric acid and 2 ml of H_2_O_2_ (30%). The digested samples were diluted to a total volume of 50 ml with ultrapure water and transferred into new tubes before analysis by using an inductively coupled plasma-mass spectrometer (ICP-MS, ELAN DRC-e).

To analyze the relative Na^+^ exclusion, Ten-day-old WT and *GmPIP1;6*-Oe transgenic soybean plants in hydroponics were treated with 100 mM NaCl for 7 days. Soybean plants after treatment were transferred into narrow neck flask individually, which filled with same volume of normal nutrient solution, and cultured for 24 hours. Na^+^ concentration of solution was measured by ICP-MS described above. The relative Na^+^ exclusion was calculated by the formula: relative Na^+^ exclusion = (Na^+^ concentration of solution × Volume of solution)/(Na^+^ concentration of shoot × DW of shoot).

### Root hydraulic conductance

Root hydraulic conductance were measured with a hydraulic conductance flow meter (HCFM) (Dynamax, Houston, TX, USA) as described in Vandeleur [23]. 5-week-old potted soybean plants grown in greenhouse. 1 day before root hydraulic conductance were measured, control plants were irrigated with normal nutrient solution and treatment plants were irrigated with nutrient solution containing 50 mM NaCl. Measurements were made between 10:00 AM to 12:00 AM. Hydraulic conductance (*L*_o_) was normalized by dividing total root dry weight. The soil was washed from the roots, and baked at 80°C for 3 days.

## Competing interests

The authors declare that they have no competing interests.

## Authors’ contributions

HS and ST conceived and designed the research. LZ, CW, RL, RV, JD and QH conducted the experiments and analyzed the data. LZ, JD and CW wrote the manuscript. All authors read and approved the manuscript.

## Supplementary Material

Additional file 1: Figure S1 Phylogenetic analysis of GmPIP1s and other AQPs by MEGA 5.04. **Figure S2.** Expression pattern of *GmPIP1;6* under NaCl treatment in leaves relative to control.. Ten-day-old soybean seedlings were treated with or without 100 mM NaCl in nutrient solution. RNA was extracted from the leaves of these seedlings at 6 hours, 12 hours, 1 day, 3 days, 5 days after treatment. All data are means of four biological replicates with error bars indicating SD. Expression level of treated plants was relative to control plants at each time point. **Figure S3.** Detection of transgenic soybean with herbicide Liberty. One half of the leaf was painted with 135 mg/L Liberty®, the bar-containing positive transgenic soybean leaves were green and the negative ones were yellow and wilted. Treated leaves were labeled with marker pen which can be seen in the images. **Figure S4.** Measurement of stomata aperture water loss rate. Ten-day-old WT and *GmPIP1;6* overexpression plants in nutrient solution were treated with or without 100 mM NaCl for 3 days. Leaves were sampled at 2:00 PM to observe the abaxial leaf surface with microscope and measured stomata aperture. Bar = 100 nm. **Figure S5.** Distribution of intracellular Na^+^ in WT and transgenic soybean plants. Ten-day-old WT and *GmPIP1;6* overexpression plants in nutrient solution were treated with or without 100 mM NaCl for 2 days. Samples of leaves were sliced and stained with CoroNa-Green at 2:00 PM and observed with a confocal microscope. Bar = 100 μm. **Figure S6.** Phenotypic characterization of *GmPIP1;6* overexpressing soybean seeds. Mature dried seeds from WT and *GmPIP1;6*-Oe transgenic soybean plants were recorded. Bar = 3 cm. Click here for file

Additional file 2: Table S1 Primers used in this study. Click here for file
