# Supplementary figures and images for: Constitutive overexpression of soybean plasma membrane intrinsic protein GmPIP1;6 confers salt tolerance
Source: BMC Plant Biol. 2014 Jul 7;14:181. doi: 10.1186/1471-2229-14-181 (PMC4105146; doi:10.1186/1471-2229-14-181)

Figure S1

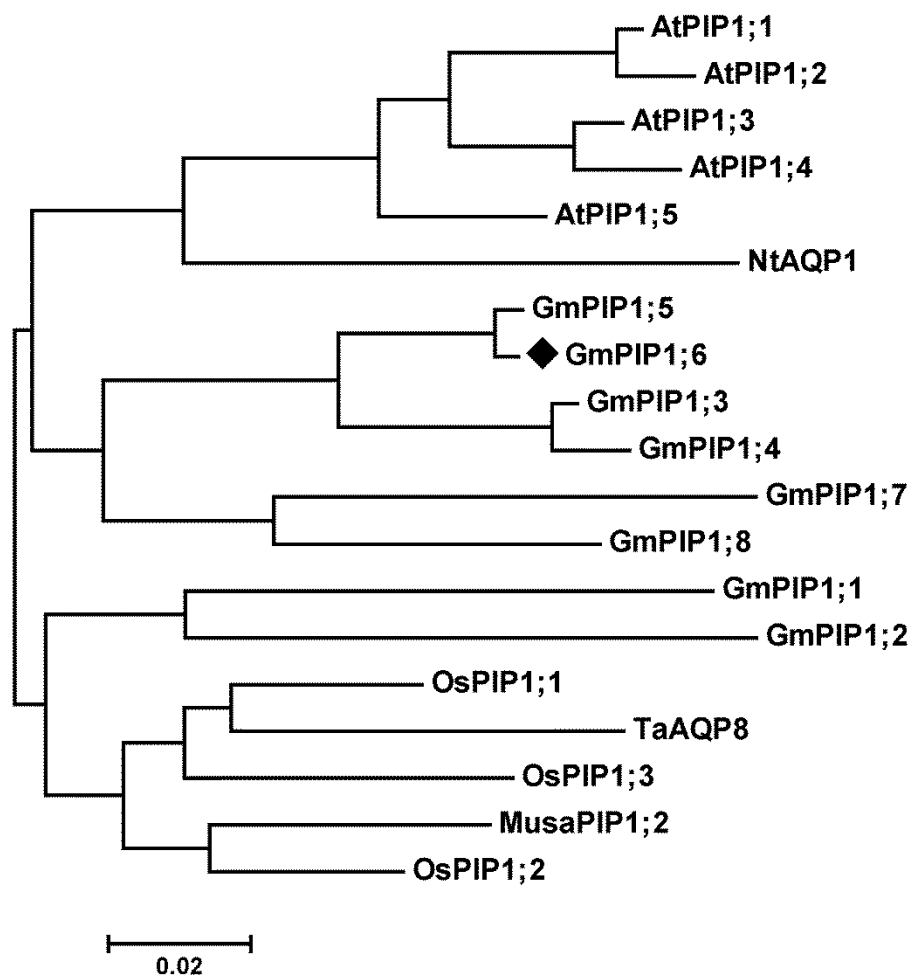

**Figure S2**

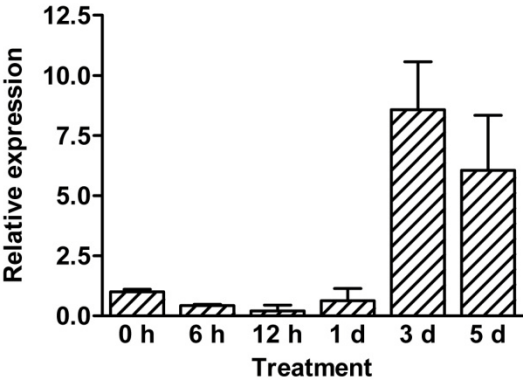

**Figure S3**

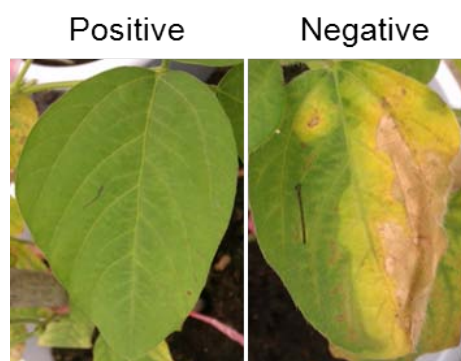

Figure S4

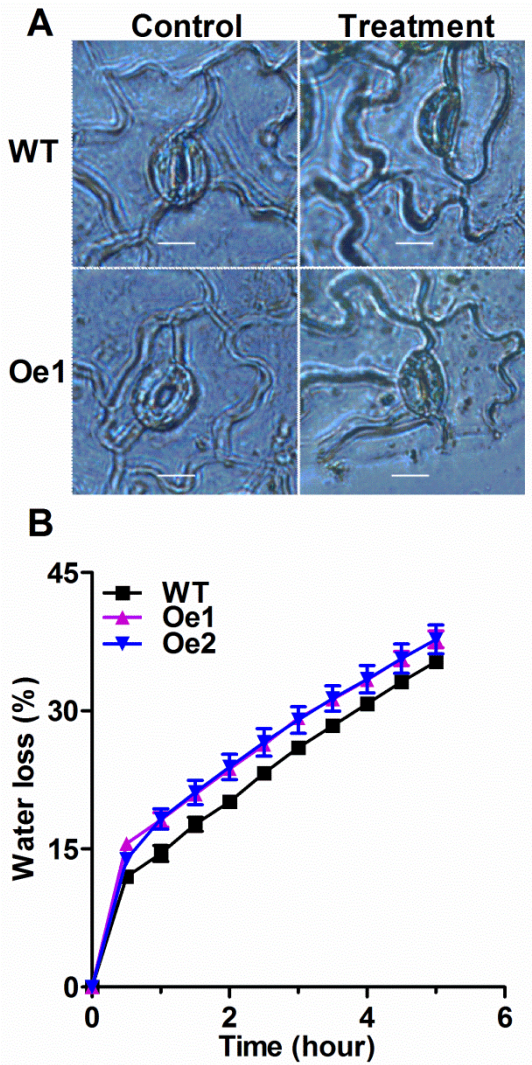

**Figure S5**

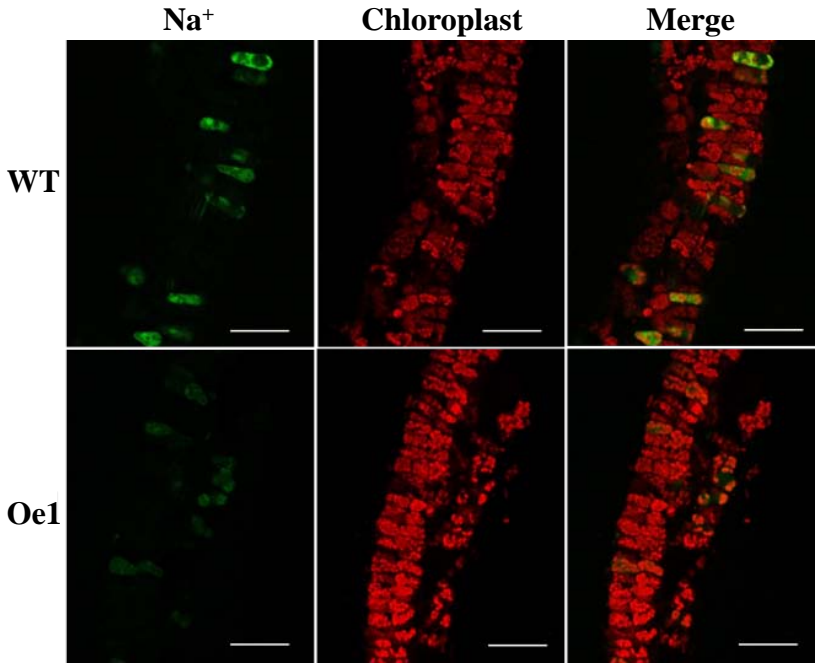

**Figure S6**

**A**

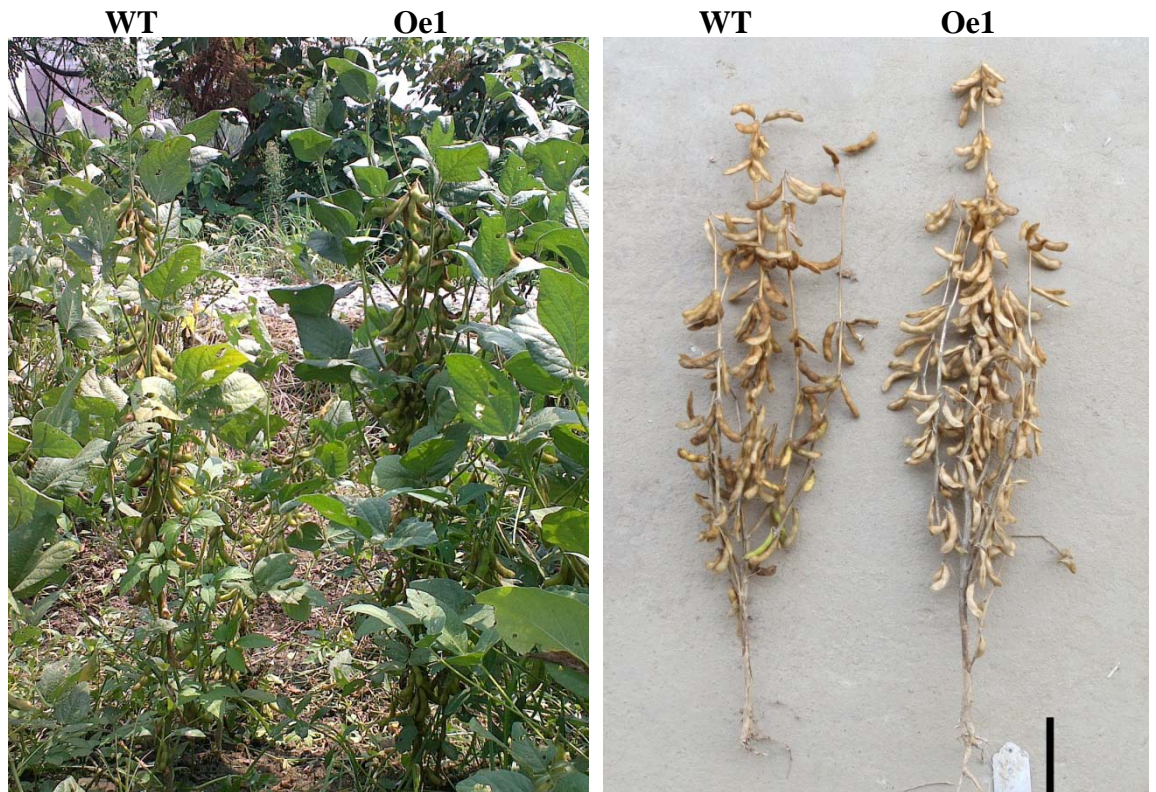

**B**

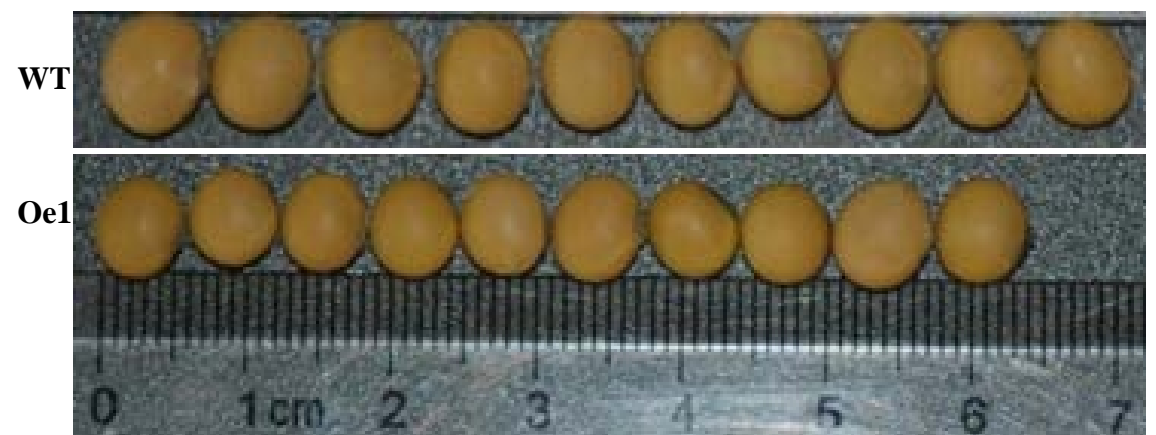

Supplement: Additional file 1: Figure S1 — Phylogenetic analysis of GmPIP1s and other AQPs by MEGA 5.04. Figure S2. Expression pattern of GmPIP1;6 under NaCl treatment in leaves relative to control.. Ten-day-old soybean seedlings were treated with or without 100 mM NaCl in nutrient solution. RNA was extracted from the leaves of these seedlings at 6 hours, 12 hours, 1 day, 3 days, 5 days after treatment. All data are means of four biological replicates with error bars indicating SD. Expression level of treated plants was relative to control plants at each time point. Figure S3. Detection of transgenic soybean with herbicide Liberty. One half of the leaf was painted with 135 mg/L Liberty®, the bar-containing positive transgenic soybean leaves were green and the negative ones were yellow and wilted. Treated leaves were labeled with marker pen which can be seen in the images. Figure S4. Measurement of stomata aperture water loss rate. Ten-day-old WT and GmPIP1;6 overexpression plants in nutrient solution were treated with or without 100 mM NaCl for 3 days. Leaves were sampled at 2:00 PM to observe the abaxial leaf surface with microscope and measured stomata aperture. Bar = 100 nm. Figure S5. Distribution of intracellular Na+ in WT and transgenic soybean plants. Ten-day-old WT and GmPIP1;6 overexpression plants in nutrient solution were treated with or without 100 mM NaCl for 2 days. Samples of leaves were sliced and stained with CoroNa-Green at 2:00 PM and observed with a confocal microscope. Bar = 100 μm. Figure S6. Phenotypic characterization of GmPIP1;6 overexpressing soybean seeds. Mature dried seeds from WT and GmPIP1;6-Oe transgenic soybean plants were recorded. Bar = 3 cm. [file 1471-2229-14-181-S1.pdf]
